# Microbial partner (MiPner) analysis

**DOI:** 10.3389/frmbi.2024.1500798

**Published:** 2025-02-07

**Authors:** Jeffrey L. Bennetzen, Josue Fernandez-Canela, Vienna Elmgreen, Shaugnessy R. McCann, Mary E. Norris, Xiangyu Deng, Philip Brailey-Crane

**Affiliations:** ^1^ Department of Genetics, University of Georgia, Athens, GA, United States; ^2^ Department of Plant Biology, University of Georgia, Athens, GA, United States; ^3^ Center for Food Safety, University of Georgia, Griffin, GA, United States

**Keywords:** microbiology, MiPner, microbe-microbe binding, microbial genomics, syncom

## Abstract

**Introduction:**

Although a few bacteria have been studied in great depth, relatively little is known about the characteristics of microbe-microbe interactions that occur within ecosystems on a daily basis. A simple, robust technique was developed to set up the foundation for investigating pairwise bacterial-bacterial interactions, using cell-cell binding as a self-selective mechanism to identify interesting bacterial species pairs.

**Methods:**

Using a *Serratia marcescens* strain (SMC43) isolated from Georgia soil as a “bait”, specific bacteria were purified by their specificity in binding SMC43 bacteria that were themselves attached to a wooden applicator stick.

**Results:**

The isolated Microbial Partners (MiPners) were greatly enriched for members of the genera *Sphingobium* and *Caulobacter*. Two streaked MiPners were unable to grow on the plates employed after separation from SMC43to be separated from, and grow on the plate type tested without, SMC43.

**Discussion:**

This suggests that the MiPner technology will be one strategy for purifying bacteria that were previously recalcitrant to culturing.

## 1 Introduction

All organisms pursue their life histories in the presence of other biological forms, some as competitors, some in a prey-predator relationship, some as co-operators, including symbionts. Microbes, in particular, commonly exist in complex communities, surrounded by hundreds to millions of other species of microbes, in such diverse and unstable environments as the atmosphere, bodies of water, multicellular hosts, and the soil. Despite this known ubiquitous and dynamic complexity, traditional microbiological research has concentrated on the study of one purified microbial species at a time to conduct hypothesis-based research, where the effects of a single variable are assayed in the presence of overall constancy. The advent and fabulous ongoing enrichment of “OMICS” technologies over the last 30 plus years (Adams et al., 1991; Patterson and Aebersold, 2003; Weckwerth, 2003; Venter et al., 2004) has provided a fully reversed perspective from the “one microbe at a time” strategy to an “everything at once” approach. This has facilitated a rapid expansion in the study of microbial ecology, especially to examine whole community microbiome interactions and to explore multi-taxa ecosystem-level interactions (Morales and Holben, 2011; Crandall et al., 2020; Chaudhry et al., 2021). The great wealth of data from OMICs approaches can provide deep and detailed correlations but come with their own weaknesses and limitations to interpretation. For instance, the enormous quantities of data and number of comparisons that are made always present statistical challenges, such as low resolving power and routine false positives, that must be rectified (Storey and Tibshirani, 2003; Carr et al., 2019). Hence, any correlations resulting from such analyses require further “hypothesis-driven” validation. With microbes, a more realistic environment for such confirmation experiments would require more than just the participation of a single microbial species.

In recent years, many research groups have attempted to create reproducible (that is, somewhat stable) microbial communities that are simpler versions of real-world assemblages (Großkopf, 2014; Mee et al., 2014; Niu et al., 2017; Zengler et al., 2019; McClure et al., 2020; Coker et al., 2022; Yin et al., 2022; Martins et al., 2023; van Leeuwen et al., 2023). The problems that must be overcome include microbial competition, different growth rates, antimicrobials, incompatible metabolic properties, and dissimilar environmental requirements (Johns et al., 2016). We propose that a simpler type of relatively stable microbial community can be created with self-identifying microbial partners. The simplest of these partnerships, with two members, would be tremendously less complex than the real world, but much more complex than a single species experiment. In understanding how microbial species interact, it is unlikely that we will be able to fully conceptualize a complex natural environment until we begin to understand binary microbial interactions.

Such binary studies do exist, though many are consigned to the exploration of syntrophic interactions in co-culture (Shou et al., 2007). However, some have provided field-relevant and fascinating information regarding microbial competition as a tool for resistance against root diseases (Thomashow, 1996), for explaining how a soil bacterium can protect a soil fungus (Dahlstrom and Newman, 2022), or in the formation, function and/or destruction of biofilms (Banks and Bryers, 1991; Nielsen et al., 2000; Breugelmans et al., 2008). These previous examples all shared intense pursuits by dedicated research teams to investigate the biology of an “important” microbe. We believe that all microbes are worthy of investigation, and that the most important discoveries may come from microbes that we do not currently know anything about. Hence, a more general and facile method for identifying microbial partnerships would provide a useful step forward.

Here, we present a novel method for isolating pairwise Microbial Partners (MiPners) from natural systems based on their propensity to physically associate with one another through microbe-microbe binding. Isolated partnerships can then be used to conduct hypothesis-based experiments. We have validated this method using a strain of *Serratia marcescens* isolated from Georgia soil, which was found to select and grow with only a tiny subset of the soil microbial collection, including with at least two bacterial strains that were unable to grow in the absence of its *S. marcescens* partner.

## 2 Materials and methods

### 2.1 Isolation and characterization of a Serratia marcescens strain, SMC43 (MiPner bait)

This study was designed to develop and demonstrate a technique to find paired microbial taxa that have explicit interactions with one another, in this case through cell-cell binding. Potential bait microbes were screened at the University of Georgia (UGA) as part of the course GENE4240L (“Experimental Microbiome Genetics”), which is designed to train students in the vagaries, certainties, and uncertainties of discovery science.

Through the Spring 2021 course, students isolated bacteria from soil samples collected on the UGA campus in Athens, GA (GPS coordinates: 33° 56’ 35.8’’ N, 83° 22’ 23.1’’ W). Soil suspensions were generated through mixing 25 g soil (from the top six inches) with 100 ml of phosphate buffered saline solution (PBS) from which 10^-2^, 10^-3^, and 10^-4^ dilutions were made with PBS. 100 microliters of each of the dilutions and the undiluted soil suspension were separately spread onto Soil Extract Agar (SEA, HiMedia Laboratories) plates. Plates were grown for two days at room temperature, single colonies were chosen by students, and these single colonies isolated to pure culture through several rounds of streaking and re-culturing on 0.1X Difco plates (Difco Nutrient Broth, BD Biosciences). Of the pure cultures generated through the class, one culture resulting from picking a red colony that we named C4-3 was later classified as *S. marcescens*, and thereafter referred to as SMC43. This culture was chosen as the bait for a MiPner proof-of-concept study because SMC43’s red pigmentation (associated with production of the antimicrobial prodigiosin; Lapenda et al., 2015) allows for easily distinguishing between the bait microbe and the bound microbes.

DNA was extracted from an SMC43 overnight liquid culture using a protocol for high molecular weight DNA extraction (Mayjonade et al., 2016). Libraries were prepared using the Rapid Barcoding kit (SQK-RBK004, Oxford Nanopore) and sequenced for 72 h using R9.4.1 flow cells (FLO-MIN106, Oxford Nanopore) on a GridION instrument. The genome was assembled from raw sequences using Canu (Koren et al., 2017). The contigs were circularized using Circlator (Hunt et al., 2015). The assembly was taxonomically analyzed with TYGS (Meier-Kolthoff and Göker, 2019). The genome was annotated using RAST (Aziz et al., 2008). A BRIG chart was constructed and annotated using Proksee (Grant et al., 2023).

### 2.2 Conceptualization and implementation of the MiPner identification strategy

**Figure 1:**
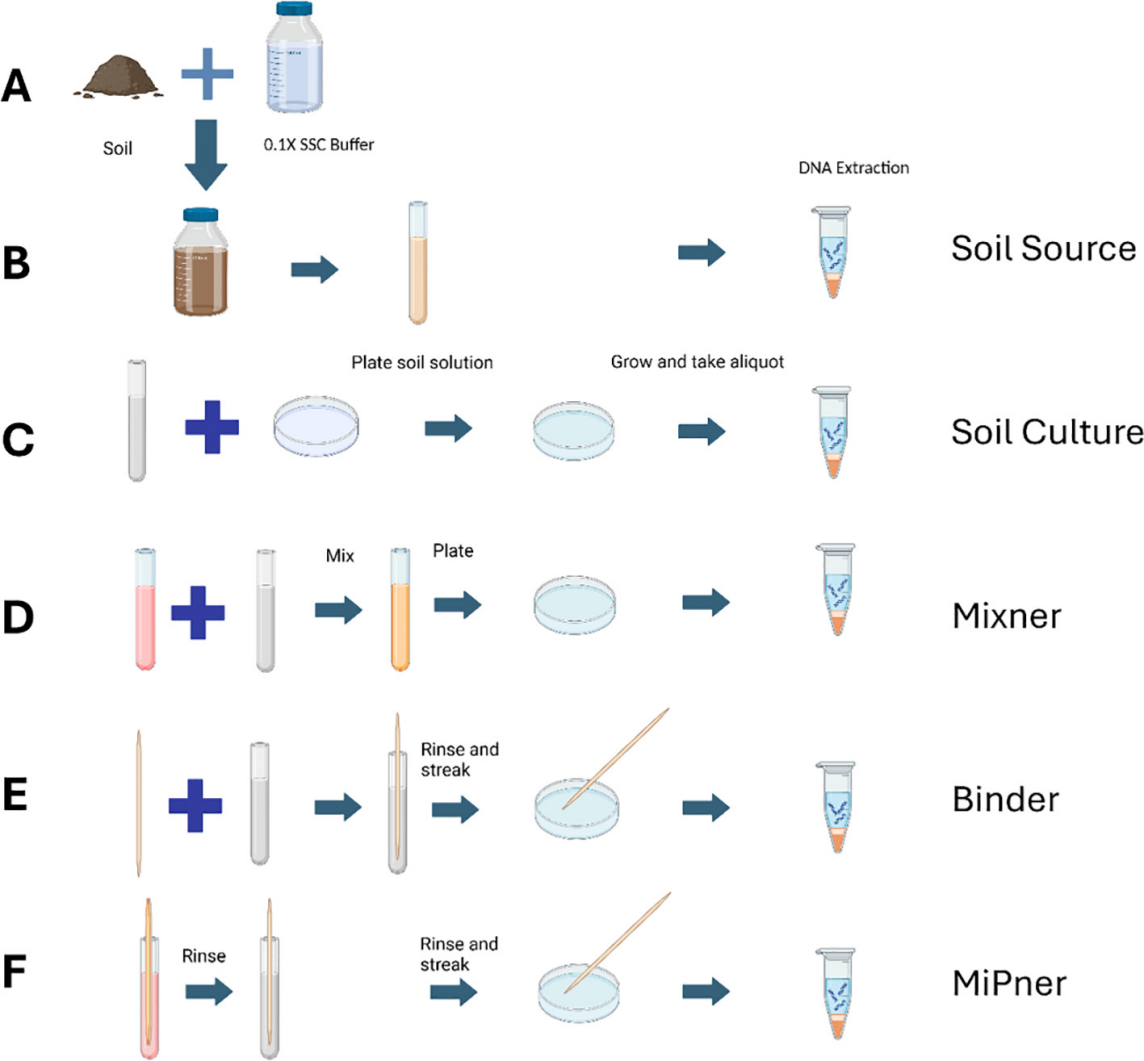
Experimental design of the MiPner strategy featuring both control experiments and the MiPner identification experiment. **(A)** Starting from soil and buffer **(B)**, a soil suspension is created from mixing the soil and buffer, allowing this to settle, and aliquoting only the top 1/3 of the suspension (Soil Source). Because this is a suspension, and continues to exhibit settling, we inverted the capped tubes gently two times prior to each use. **(C)** Microbes are plate cultured from the soil suspension (Soil Culture). **(D)** Microbes are plate cultured from the soil suspension mixed with SMC43 (Mixner). **(E)** A wooden applicator is submerged into the soil suspension and used for plate culturing of microbes (Binder). **(F)** A wooden applicator is submerged into the SMC43 solution, followed by submersion in the soil suspension, and used for plate culturing of SMC43-bound microbes (MiPner). Figure was composed using BioRender.

To isolate MiPners of SMC43, we conceived a series of steps that required both cell-cell binding of environmental bacteria to SMC43 and subsequent growth with SMC43 on Difco plates. The first component is a cultured microbial bait, in this case SMC43. The second component is a soil suspension from which MiPners can be captured through their physical associations with the bait microbe. A set of control experiments were also designed to ensure that cultured bacteria were indeed due to associations between field-sourced bacterial communities with the SMC43 bait, and not due to other factors, including lab contamination or natural binding capacity to the bait sticks used, which would result in false positives. The full MiPner and control experiment strategy is detailed in **Figure 1**.

To prepare the SMC43 bait, overnight cultures were made from pure SMC43 colonies using Difco nutrient broth held at room temperature with 150 RPM shaking. Soil solutions were made by mixing soil collected in January 2023 from the UGA campus (GPS coordinates: 33° 55’ 44.9’’ N, 83° 21’ 46.1’’ W) with 0.1X Saline-Sodium Citrate solution (15 mM NaCl, 1.5 mM Sodium Citrate, pH 7.0) at room temperature. For this soil collection, the top six inches of soil were removed and approximately 200g of soil was collected from the underlying soil using a scoopula cleaned with ethanol. The mixture was shaken at 250 rpm for 10 minutes and left for a further 10 minutes for most particulate matter to settle at the bottom of the container. The upper 25% of this suspension was decanted into a sterile 500 ml container. This soil suspension served as the microbial inoculum source for both the MiPner and control experiments (**Figure 1A**).

### 2.3 Control experiments to assess soil source community and culturing bias associated with MiPner experiments

An initial set of control experiments were conducted to both characterize the initial bacterial community present in the soil used to generate inoculations (labeled ‘Soil Source’ experimentally), and how the chosen culturing methods influence community assembly and therefore the potential for which MiPners may be identified (labeled ‘Soil Culture’ and ‘Mixner’ depending on the experiment).

The ‘Soil Source’ samples were derived from 2 ml aliquots of soil suspension described in section 2.2 (**Figure 1A**), that were centrifuged at 10,000 g for 10 minutes to precipitate bacteria and other microbes. DNA was extracted from a 250 mg pellet using a DNAeasy Power Soil Kit following the manufacturer’s protocol (Qiagen, Hilden, Germany) (**Figure 1B**). As with all samples, these preparations were prepared with three replicates. This is an important step in MiPner determination because bacteria found in the MiPner experiments but not in the source community may be from environmental contamination.

We investigated how culturing conditions could intrinsically bias the observed community when culturing both with and without SMC43. These experiments again act as controls to ensure that putative MiPners identified were indeed present within and culturable from the source community and not contaminants introduced from elsewhere. Equally important, these controls identify false negatives when compared to the soil samples, because we cannot discover any MiPner (even if it binds to our bait) by plate analysis if the MiPner will not grow on the plate. For the first culture control (labeled ‘Soil Culture’, **Figure 1C**), the soil suspension was used as an inoculant for direct culturing on 0.1 X Difco plates. 50 ul of the suspension was pipetted onto each plate across three replicates and spread using sterile glass beads. Plates were left for three days at room temperature (approximately 22°C). To capture the communities from the plates for DNA extraction, 1 ml of 0.1 X SSC was added to the plate and gently shaken for five seconds, poured off into a tube, and then used as input for DNA extraction (Hunt et al., 2015). All subsequent plates described were cultured and extracted in this manner unless otherwise specified.

The second culture control (referred to as ‘Mixner’ experiments, **Figure 1D**) was used to identify which bacteria could survive or thrive in growth with SMC43 on 0.1X Difco plates. Two experiments were performed using different ratios of SMC43 and the soil solution inputs. For the MixnerA experiments, 500 μl of the soil suspension were mixed with 500 μl of an SMC43 overnight culture in a sterile 2 ml tube. 50 μl of this mixture was spread on each of three 0.1x Difco plates with glass beads. For the MixnerB experiments, 100 μl of the soil suspension was mixed with 900 μl of a SMC43 overnight culture. As before, 50 μl of the mixture was spread onto each of three 0.1x Difco plates for culturing and DNA extraction.

### 2.4 Binder control and MiPner identification experiments

Potential false positives were identified by assessing microbes capable of directly binding to the applicator stick in the absence of SMC43 (referred to as ‘Binder’ experiments, **Figure 1E**). Any bacteria that are able to do this would be unreliable MiPners if observed in such experiments, because we would be unable to distinguish whether they were observed due to their association with SMC43 or due to their natural binding capacity to the applicator stick. Sterile wooden applicators were set in a tube containing 1 ml of soil solution for 5 minutes, and afterwards were rinsed with 0.1X SSC and then dabbed onto a fresh Kimwipe to remove the excess liquid. Each of these applicators were used to streak onto 0.1X Difco plates for culturing and DNA extraction.

The MiPner experiment entailed immersing the sterile wooden applicators first in a tube containing one ml of an overnight SMC43 culture (in 0.1X Difco, previously described) for 5 minutes. After 5 minutes, the applicators were rinsed with 0.1X SSC and then dabbed onto a fresh Kimwipe to remove excess liquid. The applicators were then immersed in a tube with one ml of soil solution. After 5 minutes, each applicator was individually rinsed with 0.1X SSC and then dabbed onto a fresh Kimwipe to remove excess liquid. Each of these applicators was used to streak onto 0.1X Difco plates for culturing and DNA extraction. A representative image of MiPner and Binder experiments can be seen in **Supplementary Figure S1** (though, in practice, these experiments were conducted in separate petri dishes).

### 2.5 Sequencing and data analysis of MiPner and control experiments

For sequencing library preparation, DNA concentration was measured fluorometrically and diluted to 2.5 ng/ul. Libraries were enzymatically fragmented and barcoded using the Plexwell 384 plate-based library preparation system (SeqWell, California), following the manufacturer’s protocol. Prepared libraries were sequenced on a NovaSeqX flow cell lane in PE150 mode (Illumina, San Diego, CA USA).

Raw sequencing reads were trimmed, and quality filtered using fastp v0.23 (Chen et al., 2018). Reads were classified using Kraken2 v2.1.3 (Wood and Salzberg, 2014) with a paired-end setup. Reads were classified against the standard Kraken2 database which draws genomes from the NCBI RefSeq database (downloaded: March 27, 2023). The relative abundances of sequences at both the genus and species level were then re-estimated using Bracken 2.7 (Lu et al., 2017). Bracken reports were merged using kraken-tools *v 1.2* (Landesman et al., 2019), and archaeal, viral and human reads were removed from the bracken reports prior to summarizing genera and species abundances.

Additional post-bracken data wrangling and figure generation was carried out with R. Diversity indices were calculated using *phyloseq* (McMurdie and Holmes, 2013) and statistically compared between experiments (source soil, soil culture and Mixners) by ANOVA. Where the ANOVA with significant *post-hoc* testing was performed with “hsd” multiple testing corrections. Upset plots were generated to display shared taxa between the same experiments using *MicrobiotaProcess* (Xu et al., 2023).

### 2.6 Sequencing and genome analysis of isolated colonies from MiPner experiments

Two distinct colonies were picked from the MiPner experiments conducted and re-cultured following methods previously described in SMC43 isolation. DNA was extracted from the pure species cultures with a protocol for high molecular weight DNA (Mayjonade et al., 2016). Libraries were prepared using the Rapid Barcoding kit (SQK-RBK004, Oxford Nanopore) and sequenced for 72 h using R9.4.1 flow cells (FLO-MIN106, Oxford Nanopore) on a GridION instrument. The genomes were assembled with Canu (Koren et al., 2017). The assemblies were taxonomically analyzed with TYGS (Meier-Kolthoff and Göker, 2019).

### 2.7 Additional MiPner experiments

Two additional MiPner experiments were performed with the same SMC43 bait and soil collected from two different sites. These experiments were not meant to investigate the control properties involved in the initial experiment, but rather to demonstrate the ease at which novel MiPners could be isolated for binding studies without any need for the controls performed in the original study. The first experiment was conducted on soil collected in July 2022 from an experimental sorghum field (33°53’25.29” N, 83°25’25.09” W) using a sterile 25 ml falcon tube, and the second was conducted from soil collected from the same location as the first MiPner experiment at a different timepoint, in September 2023, and using the same methods as described in section 2.2. Experimental methods were the same as previously described, though only the MiPner experiment itself was performed in this instance.

## 3 Results

### 3.1 Isolation and characterization of a novel strain of *S. marcescens* to use as bait

Long-read sequence information indicated that the isolated red colony is a strain of *S. marcescens*, which we refer to as SMC43. This yielded an assembled chromosome of 5,092,593 bp with a single 3071 bp plasmid. Further annotation indicated that it carried a predicted 4939 protein-encoding sequences in the chromosome and one in the plasmid (**Supplementary Figure S2**).

### 3.2 Assessment of culture bias in observed microbial community assembly

**Figure 2:**
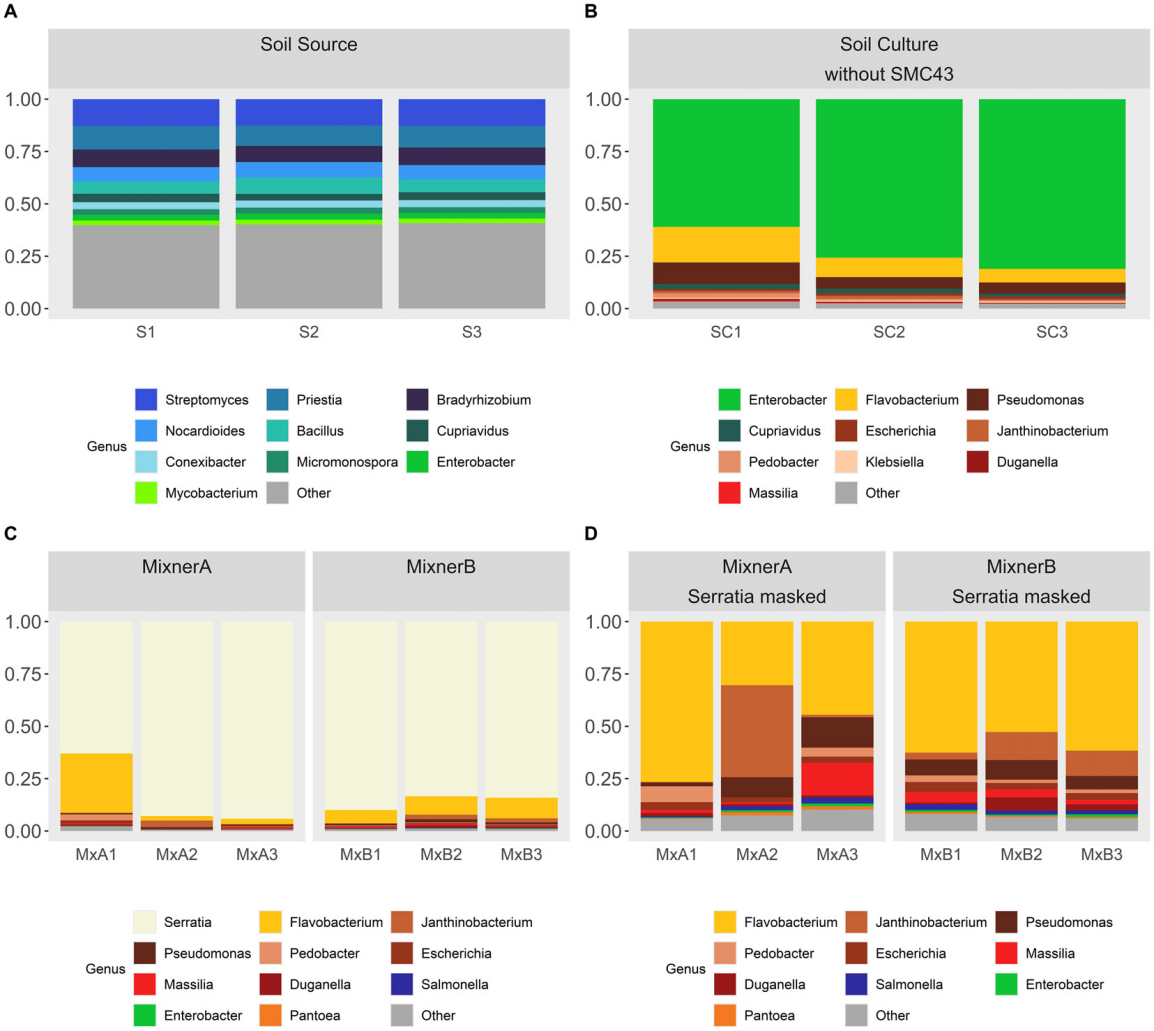
Taxa charts displaying the ten highest abundance bacterial genera identified within **(A)** the initial soil suspension and **(B)** 0.1X Difco plates inoculated with the soil suspension, **(C, D)** 0.1X Difco plates inoculated with the soil suspension and SMC43. Genera are depicted in abundance order. MixnerA and MixnerB represent plates inoculated with a ratio of 1:9 and 1:1 soil solution:SMC43 liquid culture, respectively. In panels **(C, D)**, data are presented [**(C)** without] or [**(D)** with] *Serratia* masked from the dataset so that the patterns of other bacterial abundances on the plates could be revealed more clearly. Triplicatereplicates are shown for each treatment.

Culturing soil both with and without SMC43 inoculation exerted a filtering pressure on microbial community assembly, as demonstrated through both taxonomic richness (ANOVA Species richness: F_2,9_ = 252.7, p < 0.001; ANOVA Genus richness: F_2,9_ = 497.1, p < 0.001) and diversity, assessed via Simpson’s index of diversity (ANOVA Species diversity: F_2,9_ = 25.99, p < 0.001; ANOVA Genus diversity: F_2,9_ = 9.72, p < 0.005). 522 genera in the soil source (**Supplementary Figure S3**) were completely lost through the process of culturing. The bacterial community observed in the soil solution was highly diverse, containing a mean genera richness of 672 ± 5 SE and an associated Simpson’s index of *0.95* ± 0.001 SE across the triplicate replicates. The dominant genera found within the soil solution were *Bradyrhizobium, Priestia* and *Streptomyces* with a high representation of lower-frequency microbial groups (labeled as ‘Other’, **Figure 2A**). Culturing resulted in fewer observed genera regardless of whether SMC43 was co-cultured (without SMC43: 148 ± 11.9 SE; with SMC43 MixnerA: 193 ± 32.5SE; with SMC43 Mixner B: 234 ± 14.4). In the absence of SMC43 co-culture, few genera dominated the whole community (i.e., the 10 highest abundant genera combined constituted ~ 97% of all observed reads in the three replicates without SMC43, Simpsons index = 0.45 ± 0.07SE). Culturing on 0.1X Difco plates was associated with *Enterobacter* as the dominant genus, along with moderate abundances of *Flavobacteria* and *Pseudomonas*. Genera which were highly abundant in soil solution such as *Streptomyces*, *Priestia*, and *Bradyrhizobium* did not grow as relatively well in culture (**Figure 2B**).

Co-culturing soil microbes with SMC43 resulted in only marginal, non-significant decreases in Simpson diversity relative to the soil solution extract (MixnerA: 0.62 ± 0.11SE; Mixner B: 0.63 ± 0.03SE) potentially due to the inhibition of *Enterobacter* (**Figure 2C**). Co-culture of soil microbes with SMC43 was associated with a high abundance of *Flavobacter*, *Janthinobacterium* and *Pseudomonas* (**Figure 2C**). Most Enterobacterial species were apparently killed or otherwise growth-inhibited by SMC43. The dosage of SMC43 also made a difference, with *Pedobacter* and *Xanthomonads* doing better at a lower SMC43 dosage. We use the generic name “Mixner” to denote these microbes that survive growth with our bait microbe, SMC43. Thus, Mixners could be partners in interactions with SMC43 even if they do not bind under our study conditions.

### 3.3 MiPner identification and isolation

**Figure 3:**
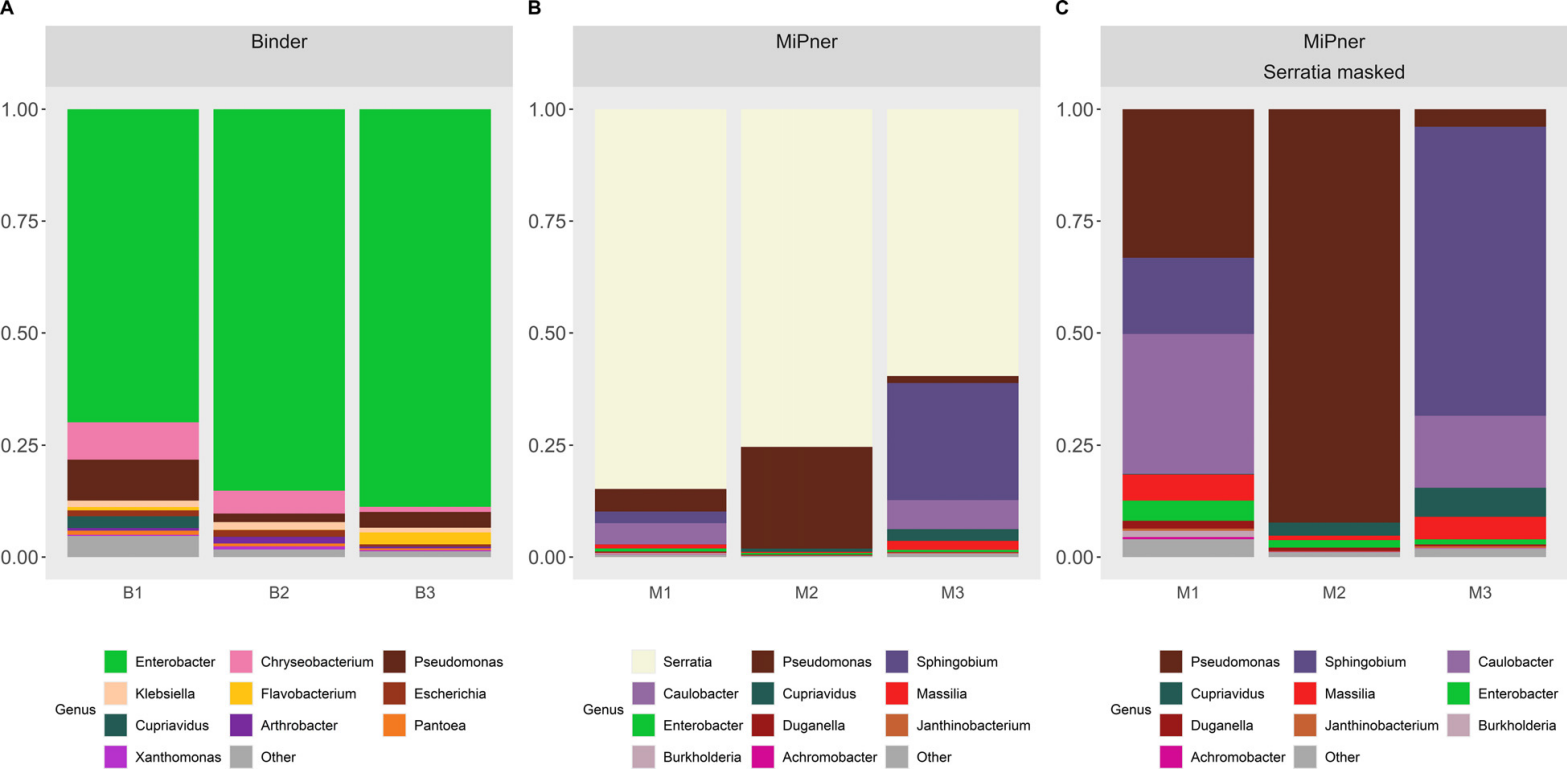
Taxa charts displaying the ten highest abundance bacterial genera observed from **(A)** the Binder experiment and **(B, C)** the MiPner experiments. Genera are depicted in abundance order. In panel **(B, C)** data are presented [**(B)** without] and [**(C)** with] Serratia masked from the dataset so that the patterns of other bacterial abundances on the plates could be revealed more clearly. Triplicate replicates are shown for each treatment.

The binding experiment was carried out both with and without SMC43 bound to the applicator stick to determine which MiPner taxa may be false positives due to their general affinity to bind to the stick surface rather than due to an explicit association with the SMC43 bait microbe (**Figure 3A**). Members of the genera *Enterobacter*, *Chryseobacterium* and *Pseudomonas* were particularly avid binders to the applicator stick under these conditions from our studied soil solution (**Figure 3A**). Through further observation of abundances at the species level (**Supplementary Data S1, S2**), we can reliably state that numerous species of both *Enterobacter* and *Chryseobacterium* were highly prevalent stick-binders. While most *Pseudomonads* can bind to the stick to varying degrees of success, there are four *Pseudomonas* species that display marked increases in their relative abundance when grown with SMC43.

There was high variability between the triplicated MiPner replicates (**Figure 3B**), a presumed outcome of the samples that were streaked on the Difco plates containing very few bacteria that passed all the requirements, especially binding to SMC43. In this case, stochastic effects could play an influential role in inter-replicate variability because of low depth in the sampling. Regardless, at least two samples contained major representation of the genera *Pseudomonas*, *Sphingobium*, and *Caulobacter* (**Figures 3B, C**). Despite its presence as the most abundant genus in the Mixner experiments (**Figure 2**), *Flavobacterium*, was not among the ten highest abundance genera in these MiPner experiments, indicating a general inability to bind SMC43 under our study conditions.

### 3.4 Genus-level enrichments across MiPner and control experiments

At the genus level, *Enterobacter* and *Pseudomonas* could not be trusted as true MiPner isolates because of their strong affinity to binding to the applicator sticks that were used (**Table 1**), despite being found in high abundance in the MiPner samples. In contrast, *Sphingobium* and *Caulobacter* were not seen to bind to the applicator at all and were rare in all other experiments, indicating at least 100-fold relative enrichment compared to the starting soil suspension. Both taxa represent genus groups that are therefore promising candidates for pursuit of further MiPner studies.

**Table 1:** Mean relative abundances per experimental condition of the ten most abundant genera identified from the MiPner experiments.

Ten Most Abundant MiPner Genera	MiPner Average (%)	Binder Average (%)	Mixner A Average (%)	Mixner B Average (%)	Soil Culture Average (%)	Soil Source Average (%)
*Pseudomonas*	43.1	4.86	8.66	7.81	7.06	0.78
*Sphingobium*	27.2	*Not present*	1.08E-03	0.01	0.01	0.26
*Caulobacter*	15.76	*Not present*	4.24E-03	0.01	0.01	0.12
*Massilia*	3.96	0.16	5.99	3.76	0.32	0.28
*Cupriavidus*	3.17	0.91	0.29	1.13	1.99	3.65
*Enterobacter*	2.44	81.27	0.88	0.94	72.56	2.75
*Duganella*	0.96	0.10	1.13	3.32	0.49	0.06
*Burkholderia*	0.49	2.27E-03	0.03	0.08	0.04	0.57
*Bradyrhizobium*	0.38	3.76E-04	0.01	0.01	6.26E-04	8.17
*Janthinobacterium*	0.35	0.17	15.11	9.60	1.09	0.04

Relative abundances are displayed across both the MiPner experiments and associated control experiments.

### 3.5 Species-level enrichments across MiPner and control experiments

At the species assignment-level, 33 individual taxa were identified belonging to the *Serratia* genus within our MiPner and control experiments. *S. marcescens* was identified in expectedly high relative abundances across the experiments where SMC43 was added (MiPner: 64.24% ± 6.35; Mixner E+F: 79.18% ± 3.63, **Supplementary Data S3**), and in low abundances within the binder control (0.01% ± 0.01) and soil culture where SMC43 was not inoculated (0.09% ± 0.004). *S. marcescens* was not observed to be present in any soil samples. Of the 32 *Serratia* taxa not attributed to *S. marscescens*, only four were found in experiments where SMC43 was not added, and when recovered in such samples, they were in low abundance (<0.01% in any relative abundance).

Relative abundances of the remaining taxa were calculated without *Serratia*. The top 100 most abundant species-level taxa across the MiPner experiments were considered to determine which individual taxa could be regarded as putative MiPners based on their presence in the MiPner experiment and their absence in the ‘Binder’ control experiment (**Supplementary Data S4**). 18 taxa were identified with > 1% relative abundance in at least one MiPner experiment sample, of which 6 were identified as putative MiPners, and 12 could not be assigned MiPner status due to their (albeit low level) presence in the binder control experiments. Putative MiPners include *Spingobium yanoikuyae, Caulobacter segnis, Sphingobium* sp. *PAMC28499, Sphingobium* sp. *LF-16, Caulobacter vibrioides* and *Burkholderia thailandensis.* Of the 12 taxa that could not be confidently assigned MiPner status with high abundances, there were assigned species of 6 *Pseudomonas*, 3 *Masilia, 1 Cupravidus, 1 Duganella*, and 1 *Enterobacter*.

21 additional putative MiPners were identified at a low relative abundance (< 1% relative abundance in at least one MiPner experiment sample, and zero abundance in the Binder control). These included several taxa that were not considered when examining genus level associations including *Bordtella parapertussis, Diaphorobacter polyhydroxybutyrativorans, Nordella* sp. *HKS 07, Pectobacterium carotovorum, Lautropia mirabilis* and *Yersinia Pestis.* Additionally, there were 5 species within the genera displayed in **Table 1** that could individually be described as putative MiPners- *Caulobacter*, 4 *Sphingobium*, 2 *Bradyrhizobium*, 2 *Masilia* and 2 *Pseudomonas* species. The detection of Pseudomonads as putative MiPners in contrast to our genus-level assessment shows that there may be some utility to considering a species level definition. As with all low-abundance taxa, however, these two identified *Pseudomonas* MiPners are unlikely to be routinely detectable through culturing efforts and, when including the high abundance (> 1%) Pseudomonads detected, represent only two out of 36 observed taxa in this genus.

### 3.6 Putative MiPner isolation, genomic and phylogenetic assessment

Genome sequences were obtained from two putative MiPner colonies. The sequences indicated that one was a strain of *Pseudomonas monteilii* with a 6.3 Mb genome and the other was a strain of *Enterobacter asburiae* with a 3.99 Mb genome. *P. monteilii* was not one of the *Pseudomonas* species that bound strongly to the applicator stick and so may represent a true positive MiPner but would require further verification in future studies. *E. asburiae* was a strong stick binder (**Supplementary Data S4**, line 28), so it seems likely that the *E. asburiae* that we isolated was not actually a true MiPner that bound strongly to SMC43.

### 3.7 Additional putative MiPner identification from subsequent experiments

In further MiPner experiments, three additional putative SMC43-associated MiPners have been identified and sequenced across two contrasting sites. From two sorghum-associated soil samples one pure putative MiPner isolate culture was found each. One was found to be an ecotype of *Stenotrophomonas maltophilia* with a 4.63 Mb genome and the other was found to be a novel *Mitsuaria* species, with a 3.62 Mb genome, that was most closely related to *Mitsuaria chitinivorans*. Additional soil collection and MiPner experiments conducted using soil from a similar location to our first MiPner experiment yielded 24 candidate MiPners, of which two candidate MiPner cultures were unable to be grown on our plates in the absence of SMC43. Sequencing of one of the paired cultures revealed that, indeed, there was both one MiPner and one SMC43 genome recovered. This MiPner is a *Mitsuaria* (that we name strain 3B1D) that appears to be a previously uncharacterized species most closely related to *Mitsuaria nodu.*

## 4 Discussion

One of the most challenging problems in the study of microbe-microbe interactions in the real world is that we neither understand the micro-environments in which these interactions occur nor have we even a faint idea of the dynamics and depths of involvement of different biological participants. Attempts to create synthetic communities are praiseworthy, but are not proven to actually replicate interactions that occur in nature (Niu et al., 2017; Zengler et al., 2019; McClure et al., 2020; Yin et al., 2022). We decided to take a different starting point, an apparent interaction, and create a system that works from that beginning. Obviously, once a two-component community is generated and investigated, adding additional components (established, for instance, by seeing what uniquely binds to a pair of interacting MiPners) will be feasible (Davis et al., 2005).

### 4.1 The challenges of growing soil bacteria

As has been heavily documented (Rappé and Giovannoni, 2003; Ernebjerg and Kishony, 2012; Shade et al., 2012; Stewart, 2012; Lloyd et al., 2018), most soil bacteria have been (so far) recalcitrant to growth on plates, even though many plate types and growth conditions have been tested. Our experiments growing soil bacteria on 0.1X Difco plates at room temperature under aerobic conditions indicated a great depletion of Acidobacteria and Actinobacteria, and a great over-representation of Proteobacteria, as has been frequently shown by others (Rappé and Giovannoni, 2003). We expect that different plating conditions would yield different enrichment/depletion patterns (Davis et al., 2005; Pham and Kim, 2012; Pham and Kim, 2016). Why these microbes are recalcitrant to culturing is not known, but it is expected to be associated with an unknown necessary component or components of their micro-environments (Pham and Kim, 2012). Perhaps one of these unknown components is a microbial partner (Burmølle et al., 2009), as manifested in our SMC43-*Mitsuaria*-3B1D result. Syntrophic interactions are not uncommon in microbial networks, of which this interdependence and need for co-culture may be an example (Dillon and Dillon, 2004). We have seen other such “cannot grow without the bait” examples from MiPner experiments with other bait species (unpublished), so it is possible that MiPner technology may be one general tool for future isolation of such recalcitrant microbes.

Of course, our SMC43-associated *Mitsuaria* may be able to grow alone on some plate types if we pursued a full round of investigations. Many *Mitsuaria* grow on several different plate types, as we have seen in our lab, including 0.1X Difco plates (Fan et al., 2018), but we believe it is likely that many such future “bait-requiring” microbe isolations will be of species that have an absolute partner requirement. Regardless, the SMC43-*Mitsuaria*-3B1D interaction on 0.1X Difco plates indicates a pairwise interaction that is obligate for this *Mitsuaria*’s growth, and future studies showing what SMC43 provides in this relationship will be of great interest.

### 4.2 Challenges associated with molecular taxonomic identification

The limited presence of *S. marcescens* in binder and soil culture samples could be due to either incredibly low-level cross-contamination of these samples during the experiments and/or sequence prep, or likely due to levels of *S. marcescens* (and other related species) in the soil extracts that was below the detection threshold of sequencing in these samples but not within other experiments where diversity was artificially lowered by experimental conditions. Due to the nature of *Kraken2/Bracken* taxonomy assignment and abundance estimation, the majority of the remaining *Serratia* taxa sequence assignments are also likely derived from low-information DNA regions of the sequenced SMC43 isolate or related environmental taxa. Of the 32 *Serratia* taxa not directly attributed to *S. marcescens*, only four were found in experiments where SMC43 was not added, and when they were recovered in these samples, they were in incredibly low abundance (< 0.01% mean relative abundance in any experiment group). This demonstrates that for mixed bacterial communities, the interpretation of species-level assignments based on *Kraken2/Bracken* requires some caution. Low capacity for species resolution as demonstrated by our *Serratia* analysis, also calls into question how definitive this approach can be in comparison to genus level assessments without further culturing.

### 4.3 MiPner specificity

The different and taxonomically limited set of MiPner microbes identified, compared to our starting soil and to other selective steps (e.g., plate type) in the technique, indicates that the microbe-microbe binding is highly selective and robust enough to avoid removal by a simple rinsing step. Moreover, this binding requires only a few minutes to generate this specificity and durability. Preliminary studies in our lab using different bait species on the same soil suspension (unpublished results) have suggested that each bait generates a separate set of MiPners that are enormously enriched at the genus level. Once pursued, we expect that observed species-level binding specificities and enrichments will be even more dramatic. Hence, there is an unlimited potential for using MiPner technology to find potential interacting partners with most other microbes, and this should extend beyond just bacteria.

Isolation of colonies growing on the bait streak is not likely to only yield MiPners, as shown with our *E. asburiae* result. Anything that binds the applicator stick found in the study does not need to bind the bait, although it is required to grow with the bait on the plate type used. For this study, we would be confident of the MiPner status of any *Sphingobium* or *Caulobacter* isolated, which could be further confirmed by reciprocal binding studies.

### 4.4 The full set of potential bait partners

There is no reason to believe that any bacterium interacts with the same set of microbial partners in all environments. Our investigations of different soils with SMC43 indicated different final MiPner outcomes, dependent on soil source. The two *Mitsuaria* that we found in a subsequent experiment to the one described in detail here, represent a genus that was fully absent from all our sequencing in the first experiment. Hence, discovery of the full set of SMC43 MiPners that may be real-life microbial partners would be best pursued with a number of different soil sources. And this will be equally true for any other microbe used as bait.

### 4.5 Mixner results

We call the community DNA sequence results of growing one bait microbe with a soil suspension of microbes to be the outcome of a Mixed preMiPner, or Mixner, experiment. Our Mixner outcomes indicate variable survival patterns of the soil-solution microbes depending upon the dose of the bait microbe. Of course, SMC43 may provide a severe example of this phenomenon because of its production of prodigiosin, a potent anti-microbial (Lapenda et al., 2015). However, many bacteria produce antimicrobials, so we predict this ratio-dependence result to be generally true with any bait microbe or any mixed microbe suspension. Perhaps at lower bait dosages, other microbes have a greater opportunity to build communities that will resist any negative (or positive) contributions from the bait microbe. In the effort to identify binding partners, it is unrealistic at this stage to try to find every microbe that might bind a specific bait. Hence, the false negatives suggested by Mixner experiments do not impede one’s ability to find a wealth of real positives in MiPner binding studies. Hence, Mixner controls should not be necessary in routine MiPner analyses.

### 4.6 MiPner enrichment

Several steps of enrichment led to the isolated microbes that were dubbed MiPners. The choice of a liquid suspension, rather than total soil, as the initial soil microbe source was one such enrichment/depletion step. As noted, growth on a plate and survival of exposure to SMC43 were other selection steps, each with unique outcomes. The primary goal of this technology, and thus the most interesting enrichment for us, is to find paired candidates for a specific SMC43-MiPner interaction. Starting with these functional components of a durable and highly-specific binding, study of these two-species interactions can proceed in a wealth of directions. Characterization of each of these pairs of interactions are warranted by such techniques as annotation for gene-enrichment by the selection process, optical studies of the physical interaction, forward genetic searches for genes that decrease or increase the partnership, reverse genetics of genes likely to be involved in the binding and other interactions, transcriptomic/proteomic/metabolomic analysis of inductions/repressions by the interactions, and many others too numerous to list. All of these are beyond the scope of our current investigation.

It should be noted that none of the controls that we pursued in this study would be necessary to pull out MiPners that bound to the bait. Just the simple bait binding to an applicator, followed by the second immersion and plating, would be sufficient to find a candidate partner. However, the various controls were of value in determining the likelihood that the identified microbes truly were MiPners, especially in distinguishing microbes that bound to the applicator stick without bait involvement. Full confirmation of a MiPner would be best pursued by subsequent studies, especially by using the MiPner as bait to see if it reciprocally pulls out its partner that was the initial bait.

### 4.7 MiPner generality

As should be clear, there is absolutely nothing about the MiPner strategy that is limited to any specific bait microbe or to any specific microbial community. The animal gut (Dillon and Dillon, 2004; Gill et al., 2006; Hitch et al., 2022) or bodies of water (Venter et al., 2004; Diao et al., 2017) and such fascinating microbial worlds as permafrost (Wu et al., 2022), the digestive fluids of pitcher plants (Zhang et al., 2020a; Zhang et al., 2020b) or waste tailings (Yagi et al., 2009) will be equally accessible to this technology. There is potential for viruses, fungi, protists, tiny invertebrates or archaea to be used as baits or followed as MiPners. Moreover, it is difficult to overestimate the speed, simplicity, robustness and low cost of this approach. We hope that many laboratories will join us in MiPner experimentation, so that we can begin to assemble a microbial interaction atlas, starting with two species at a time.

## Data Availability

The original contributions presented in the study are publicly available. This data can be found here: NCBI, accession PRJNA1201191. Kraken2-derived count tables can be found in the **Supplementary Material**.
