# Supplementary material for: Microbial partner (MiPner) analysis
Source: Front Microbiomes. 2025 Feb 7;3:1500798. doi: 10.3389/frmbi.2024.1500798 (PMC12993605; doi:10.3389/frmbi.2024.1500798)
Supplement: Supplementary file 1 [file DataSheet1.zip › Supplementary Figures.docx]

# SUPPLEMENTARY MATERIALS

### **
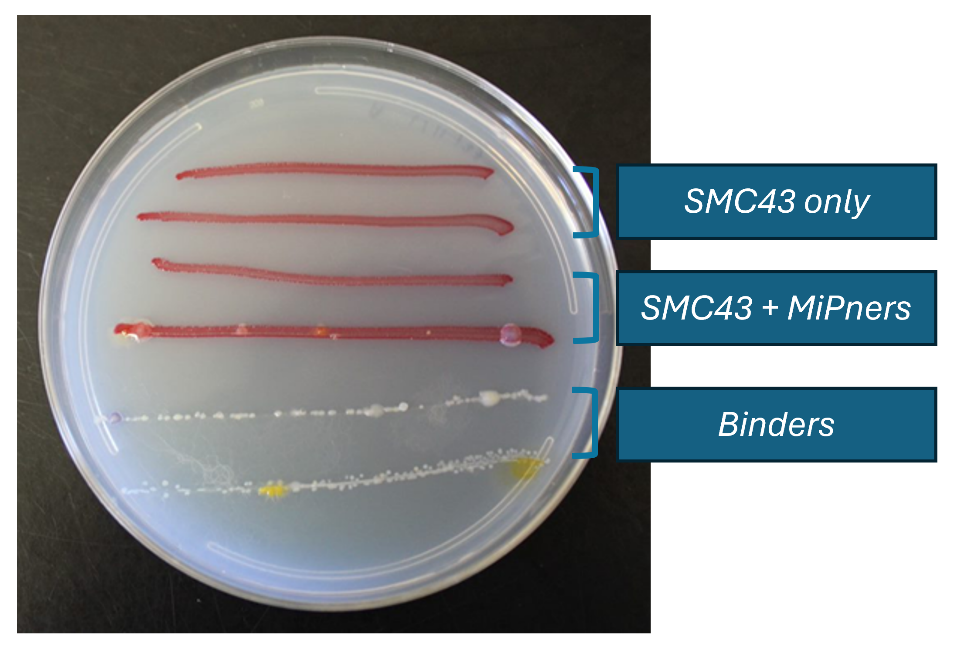
**

### **Figure S1.** A demonstrative 0.1X Difco plate grown for three days at room temperature with streaks of SMC43 (lines 1, 2), streaks of SMC43 plus putative MiPners (lines 3, 4) and with streaks of microbes that bound to the applicator stick in the absence of SMC43 pre-binding (lines 5, 6).


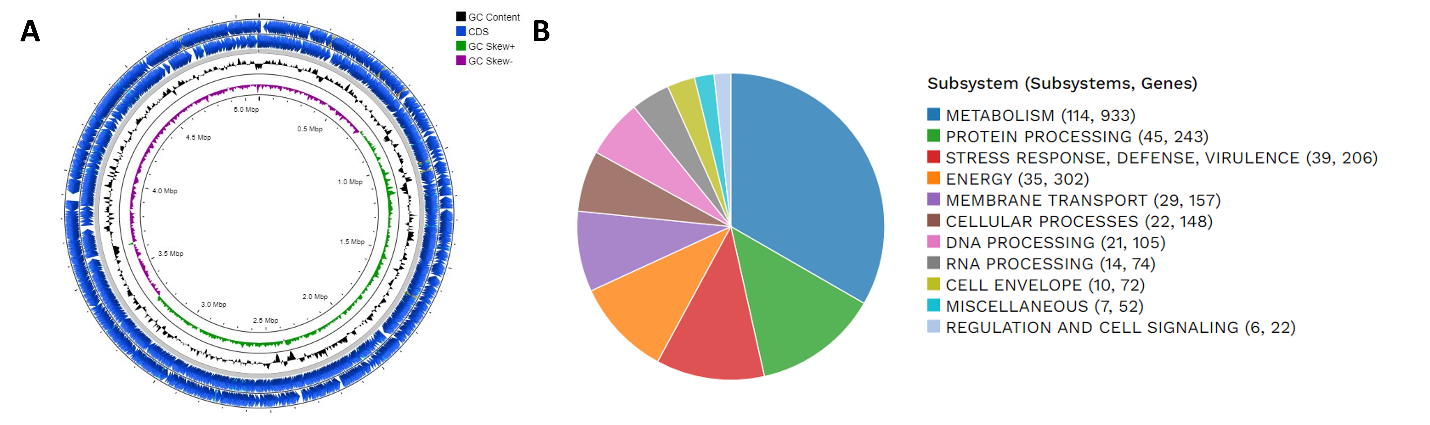


### **Figure S2.** A) Genomic map and BRIG analysis of SMC43, featuring the circular map of the genome. Outer circle to inner circle (CDS, GC content, GC skew+, GC skew -, and GC), B) Subsystem distribution based on RAST SEED analysis of SMC43. The pie chart organizes cellular processes. The number of protein-coding genes predicted to be involved in each cellular process are indicated in parentheses.


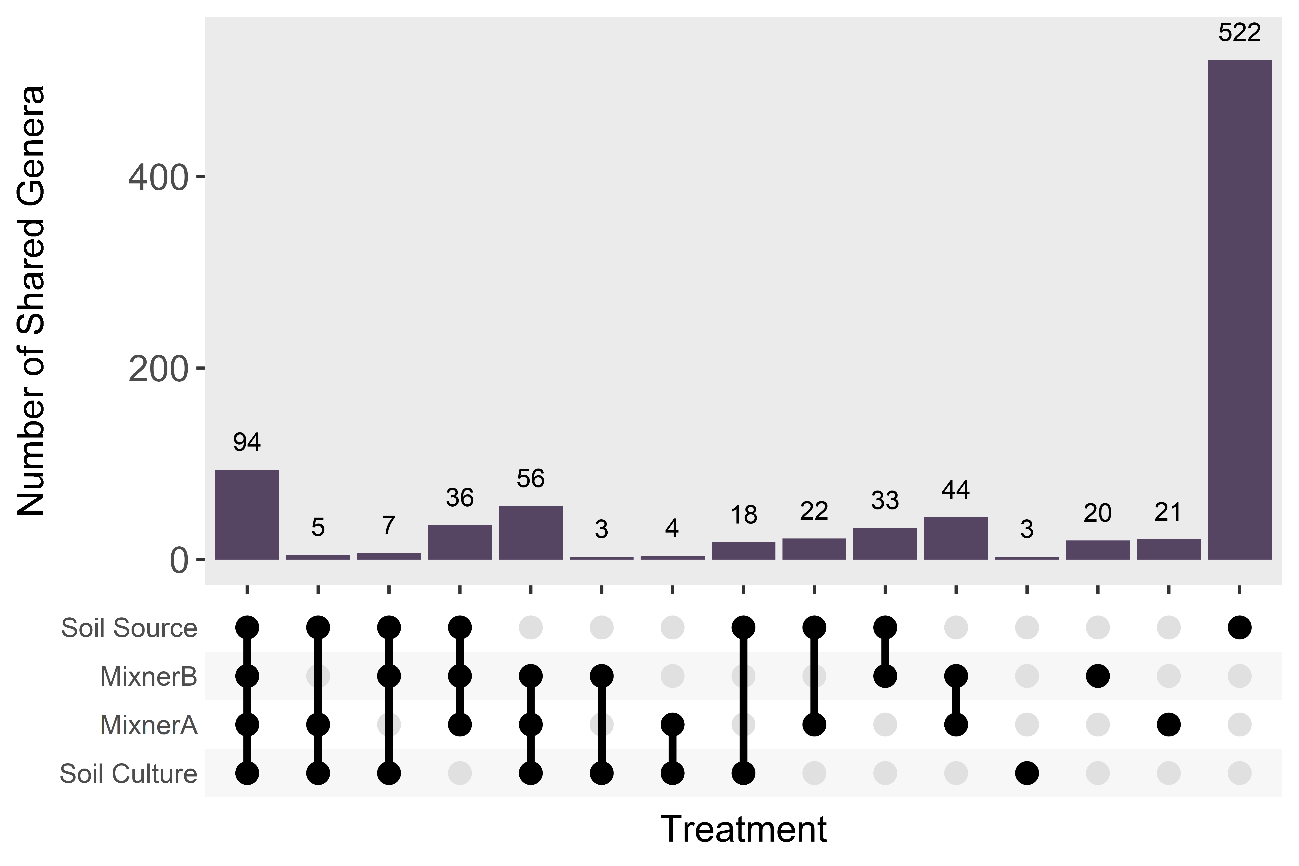


***Figure S3.*** *Upset plot displaying the number of genera shared between each culture control treatments employed in this study.*
